# Impact of bench repair for donor mitral valve before orthotopic heart transplantation: a case report

**DOI:** 10.1186/s44215-023-00070-1

**Published:** 2023-05-29

**Authors:** Tsubasa Mikami, Takuji Kawamura, Yoshito Ito, Yusuke Misumi, Noriyuki Kashiyama, Ai Kawamura, Masashi Kawamura, Daisuke Yoshioka, Kazuo Shimamura, Koichi Toda, Shigeru Miyagawa

**Affiliations:** 1 Department of Cardiovascular Surgery, Osaka University Graduate School of Medicine, 2-2-E1, Yamadaoka, Suita, Osaka 565-0871 Japan; 2 Department of Cardiovascular Surgery, Fukui Cardiovascular Center, Fukui, Japan; 3 Department of Thoracic and Cardiovascular Surgery, Dokkyo Medical University Saitama Medical Center, Koshigaya, Japan

**Keywords:** Heart transplantation, Marginal donor, Mitral valve repair

## Abstract

**Background:**

The use of donor hearts with valvular disease has been considered debatable in heart transplantation for many years. However, few reports indicate successful heart transplantation using donor hearts with mitral regurgitation that underwent mitral valve repair on the back bench.

**Case presentation:**

We report two cases of a 38-year-old and a 48-year-old woman with implantable left ventricular assist devices who underwent heart transplantation at our institution. Transthoracic echocardiography of donor hearts just before the explant revealed that each donor heart had preserved cardiac function and significant mitral regurgitation due to mitral posterior leaflet prolapse and annular dilatation, respectively. Bench mitral valve repair was accomplished using triangular resection for one patient and annuloplasty for the other. This was followed by confirmation of excellent mitral leaflet coaptation without residual mitral regurgitation. Transthoracic echocardiography and right heart catheterization performed 6 months after transplantation clarified the favorable cardiac function of each transplanted heart without mitral regurgitation recurrence.

**Conclusions:**

Efficient utilization of donor hearts with mitral regurgitation may be acceptable when the cardiac function of donor hearts with mitral regurgitation is preserved and heart transplantation, including bench mitral valve repair, is feasible within an acceptable ischemic time.

**Supplementary Information:**

The online version contains supplementary material available at 10.1186/s44215-023-00070-1.

## Background

Regarding the use of donors with preexisting cardiac abnormalities, the International Society of Heart and Lung Transplantation guidelines state that anatomically abnormal aortic and mitral valves may undergo bench repair or replacement with subsequent heart transplantation (HTx) [1]. Nevertheless, for many years, donor hearts with valvular disease were generally classified as marginal and were contraindicatory for use in HTx [2–8]. On the other hand, few reports indicate HTx using donor hearts with no cardiac problems other than a valvular disease, especially mitral regurgitation (MR) [2–10]. Herein, we report two rare cases in which bench mitral valve repair (MVr) was performed on the donor hearts with significant MR before HTx.

## Case presentation

### Patient no. 1

A 38-year-old woman with an implantable left ventricular assist device (LVAD) for doxorubicin-induced cardiomyopathy awaited HTx. Meanwhile, the patient developed a pump infection and underwent an emergency extracorporeal LVAD conversion combined with omentopexy. Although her organ function was maintained thereafter, it was challenging to continue management with extracorporeal LVAD due to prolonged infection control and hospitalization.

A female donor in her 50 s experienced brain death due to hypoxic encephalopathy. The cardiac function of the donor heart was preserved (left ventricular ejection fraction (LVEF), 65%; left ventricular end-diastolic diameter (LVEDD), 45 mm; left ventricular end-systolic diameter (LVESD), 29 mm) with a low-dose inotrope (dopamine 3 μg/kg/min). However, the donor heart was declined by all other transplant programs because it contained the prosthetic vascular graft of the ascending aorta. In addition, transthoracic echocardiography (TTE) performed immediately before the explant revealed severe MR due to P2 prolapse. Given the condition of the recipient and position on the waiting list, we proceeded with the HTx.

**Fig. 1:**
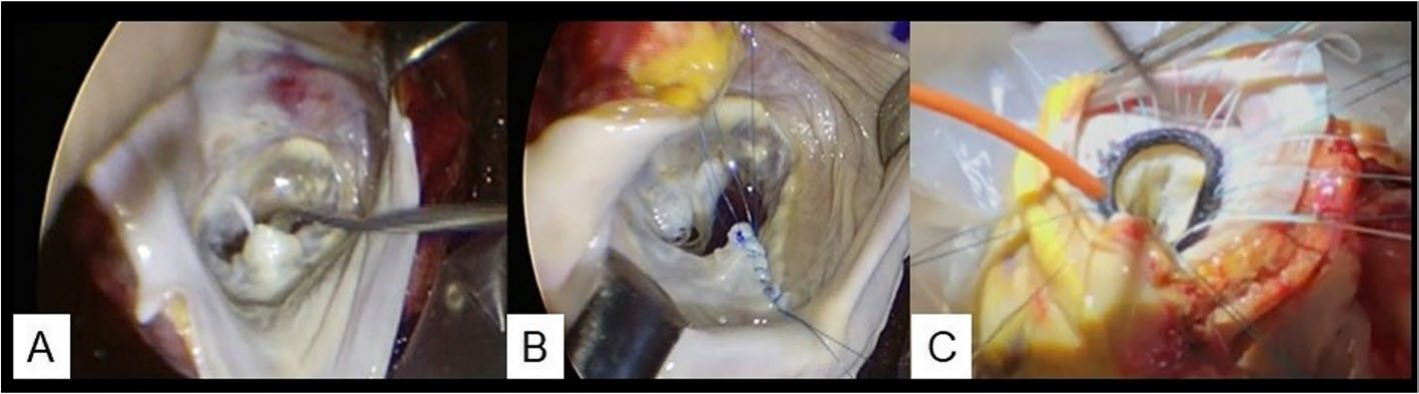
Intraoperative images of the donor heart on the back bench. The donor heart of patient no. 1 has (**A**) severe mitral regurgitation due to P2 prolapse with chordae rupture and (**B**) treated with triangular resection and suture. Patient no. 2 has functional mitral regurgitation due to annular dilatation (**C**) and is treated with mitral annuloplasty

The donor heart was transported to our institution, and the mitral valve (MV) was carefully inspected on the back bench through the open left atrium, which revealed significant mitral valve prolapse in the P2 segment with chordae rupture (Fig. 1A). Bench MVr was accomplished using triangular resection and suturing of the posterior MV leaflet on P2 segment (Fig. 1B). These procedures required an additional ischemic time of approximately 15 min. Considering the patient’s preoperative general condition, we decided not to perform mitral annuloplasty with a prosthetic ring because the patient was likely to develop bacteremia even after HTx. On saline pressure testing with a prosthetic vascular graft of the clamped ascending aorta, excellent coaptation of the mitral leaflets was confirmed by the absence of MR. Subsequently, the donor heart was implanted using a modified bicaval anastomosis technique. The total allograft ischemic time was 246 min.

The patient developed mediastinitis and aspiration pneumonia postoperatively. Owing to her poor preoperative condition, the postoperative treatment was prolonged, requiring long-term rehabilitation. Nevertheless, the transplanted heart function was stable, as confirmed by the favorable results of TTE and right heart catheterization (RHC) performed 6 months after surgery (Supplementary Table 1). However, 12 months after HTx, the patient developed aspiration pneumonia resulting in the gradual progression of organ disorders. Eventually, the patient died of multiple organ failure 19 months after HTx. No MR recurrence was observed in the transplanted heart until death.

### Patient no. 2

A 48-year-old woman with an implantable LVAD for ischemic cardiomyopathy awaited HTx. As the patient suffered from a refractory infection due to an abscess around the outflow cannula that required open drainage, elective pump exchange was considered the treatment of choice.

A female donor in her 50 s experienced brain death due to hypoxic encephalopathy. Since the cardiac function of the donor heart was preserved (LVEF, 63%; LVEDD, 39 mm; and LVESD, 26 mm) with vasoconstrictors at acceptable doses (noradrenaline 0.05 μg/kg/min and vasopressin 3 U/h), we determined to perform HTx using this donor heart. However, TTE just before the explant revealed moderate-to-severe MR, probably due to annular dilatation and volume overload. Given the condition of the recipient and the MR etiology of the donor heart, we proceeded with HTx.

The donor heart was transported to our institution, and the MV was carefully inspected on the back bench through the open left atrium. No significant problems were observed in either the MV leaflets or subvalvular mitral apparatus. Bench mitral annuloplasty was performed using a 30-mm Memo 3D ring (LivaNova, Saluggia, Italy), which required approximately 20 min of additional ischemic time (Fig. 1C). Excellent coaptation of the MV leaflets without residual MR was confirmed by saline pressure testing. Subsequently, the donor heart was implanted using the modified bicaval anastomosis technique. The total allograft ischemic time was 212 min.

Postoperatively, the patient developed pulmonary aspergillosis and subsequently suffered from aspergillosis mediastinitis due to a pulmonary fistula into the mediastinum, which was successfully treated with right middle lobectomy combined with antifungal drugs. TTE and RHC performed 6 months after surgery revealed stable cardiac function in the transplanted heart (Supplementary Table 1). The patient was stable for the following 18 months without MR recurrence in the transplanted heart.

## Discussion and conclusions

Reflecting the shortage of cardiac donors, attempts are being made to efficiently utilize marginal donors, with reported acceptable postoperative outcomes [11, 12]. Donor hearts with the valvular disease are regarded as marginal donors in the modern era. However, they have the potential to become effective resources as cardiac donors for HTx, considering the remarkable improvements in the treatment of valvular diseases in recent years [13, 14]. Similar to our cases, few reports indicate HTx using donor hearts with MR (Supplementary Table 2) [2, 4–10]. Therefore, bench MVr for donor hearts with MR is expected to be an effective method of utilizing marginal donors, as a solution to the cardiac donor shortage.

Bench MVr for donor hearts with MR has rarely been performed due to concerns regarding the feasibility and durability of MVr [6]. However, reports indicate that it is a relatively straightforward and easy technique because of the perfect exposure of the MV with a completely opened left atrium [2, 3, 7, 9]. Similar to the reported cases, in both cases, we successfully achieved durable MVr within an acceptable time. However, these results are based on the fact that neither our nor previous cases had a high MR complexity in donor hearts. Therefore, to avoid residual MR in the donor heart, the therapeutic target should be restricted to the MR in the donor heart, where MVr is considered highly feasible. Alternatively, if MVr for complex regurgitation mechanisms is required, a detailed evaluation for MR with transesophageal echocardiography should be considered prior to donor heart explant. In addition, if significant MR remains after MVr, it would be beneficial to consider in advance how to approach the left atrium and MV based on the method of HTx. Specifically, our plan was to approach the MV by partially resecting the right side of the left atrial anastomosis between the donor and recipient hearts. Furthermore, even if this did not provide a surgical field for the MV, we determined that a further resection of the superior and inferior vena cava anastomosis would provide a sufficient surgical field for the MV equivalent to a conventional left atriotomy.

When using the donor heart with MR in the HTx, minimizing the ischemic time assumes more importance, given the time required for MVr on the bench. This is because determining the exact time required for MVr is challenging because it depends on the complexity of MR, although bench MVr did not take much time not only for the previous cases but also in ours [3, 7–9]. In addition, the majority of ischemic time was actually due to travel back from the donor location [2, 8, 9]. Therefore, to suppress the ischemic time effectively, the recipient of the cardiac donor should be selected to enable shorter transport distances.

In conclusion, we reported two cases of HTx using donor hearts with MR that were successfully treated with MVr on the back bench. Efficient utilization of donor hearts with MR may be acceptable when the cardiac function of the donor heart with MR is preserved and HTx, including bench MVr, is feasible within an acceptable ischemic time.

## Supplementary Information

**Additional file 1:** **Supplementary Table 1.** Postoperative transthoracic echocardiography and right heart catheterization performed on transplanted hearts 6 months after transplantation.

**Additional file 2:** **Supplementary Table 2.** Surgical cases using donor hearts having mitral regurgitation with bench mitral valve repair before heart transplantation.

## Data Availability

The datasets generated and/or analyzed during the current study are available from the corresponding author upon reasonable request.

## Abbreviations

HTx: Heart transplantation
LVAD: Left ventricular assist device
MR: Mitral regurgitation
MV: Mitral valve
MVr: Mitral valve repair
RHC: Right heart catheterization
TTE: Transthoracic echocardiography

## References

[1] Costanzo MR, Dipchand A, Starling R, Anderson A, Chan M, Desai S, et al. International society of heart and lung transplantation guidelines. . the international society of heart and lung transplantation guidelines for the care of heart transplant recipients. J Heart Lung Transplant. 2010;29:914–56. 20643330 10.1016/j.healun.2010.05.034

[2] Massad MG, Smedira NG, Hobbs RE, Hoercher K, Vandervoort P, McCarthy PM. Bench repair of donor mitral valve before heart transplantation. Ann Thorac Surg. 1996;61:1833–5. 8651799 10.1016/0003-4975(96)00093-8

[3] Antunes PE, Prieto D, Eugénio L, Antunes MJ. Donor mitral valve repair in cardiac transplantation. J Thorac Cardiovasc Surg. 2005;129:227–8. 15632854 10.1016/j.jtcvs.2004.04.041

[4] Prieto D, Antunes P, Antunes MJ. Donor mitral valve repair in cardiac transplantation. Transplant Proc. 2009;41:932–4. 19376391 10.1016/j.transproceed.2009.01.060

[5] Pawale A, Tang GH, Milla F, Pinney S, Adams DH, Anyanwu AC. Bench mitral valve repair of donor hearts before orthotopic heart transplantation. Circ Heart Fail. 2012;5:e96–7. 23170027 10.1161/CIRCHEARTFAILURE.112.970962

[6] Okamura H, Woo YJ. Ex vivo allograft mitral valve leaflet repair prior to orthotopic heart transplantation. J Card Surg. 2014;29:424–6. 24460568 10.1111/jocs.12297

[7] Sprengel A, Skwara W, Ziegelhöffer T, Cetinkaya A, Schönburg M, Richter M. Combined mitral valve repair and heart transplantation. Clin Case Rep. 2018;6:564–8. 29636914 10.1002/ccr3.1342 PMC5889224

[8] Fiore A, Grande AM, Gatti G, Youssari A, Piscitelli M, Bergoend E, et al. Valvular surgery in donor hearts before orthotopic heart transplantation. Arch Cardiovasc Dis. 2020;113:674–8. 32868256 10.1016/j.acvd.2020.05.010

[9] Risher WH, Ochsner JL, Van Meter C. Cardiac transplantation after donor mitral valve commissurotomy. Ann Thorac Surg. 1994;57:221–2. 8279899 10.1016/0003-4975(94)90406-5

[10] Michler RE, Camacho DR. Ex-vivo mitral valve repair prior to orthotopic cardiac transplantation. Ann Thorac Surg. 2002;73:962–3. 11899212 10.1016/s0003-4975(01)02865-x

[11] Huckaby LV, Hickey G, Sultan I, Kilic A. Trends in the utilization of marginal donors for orthotopic heart transplantation. J Card Surg. 2021;36:1270–6. 33484206 10.1111/jocs.15359 PMC9122040

[12] Trivedi JR, Cheng A, Ising M, Lenneman A, Birks E, Slaughter MS. Heart transplant survival based on recipient and donor risk scoring: a UNOS database analysis. ASAIO J. 2016;62:297–301. 26771395 10.1097/MAT.0000000000000337

[13] Baumgartner H, Falk V, Bax JJ, De Bonis M, Hamm C, Holm PJ, et al. 2017 ESC/EACTS Guidelines for the management of valvular heart disease. Eur Heart J. 2017;38:2739–91. 28886619 10.1093/eurheartj/ehx391

[14] Otto CM, Nishimura RA, Bonow RO, Carabello BA, Erwin JP 3rd, Gentile F, et al. 2020 ACC/AHA Guideline for the Management of Patients with Valvular Heart Disease: executive summary: a report of the American College of Cardiology/American Heart Association Joint Committee on Clinical Practice Guidelines. Circulation. 2021;143:e35–71. 33332149 10.1161/CIR.0000000000000932

