# Supplementary material for: Impact of bench repair for donor mitral valve before orthotopic heart transplantation: a case report
Source: Gen Thorac Cardiovasc Surg Cases. 2023 May 29;2:61. doi: 10.1186/s44215-023-00070-1 (PMC11533544; doi:10.1186/s44215-023-00070-1)
Supplement: Supplementary file 1 — Additional file 1: Supplementary Table 1. Postoperative transthoracic echocardiography and right heart catheterization performed on transplanted hearts 6 months after transplantation. [file 44215_2023_70_MOESM1_ESM.docx]

**Supplementary Table 1**. Postoperative transthoracic echocardiography and right heart catheterization performed on transplanted hearts 6 months after transplantation

| Patient | **transthoracic echocardiography** | | | | **right heart catheterization** | | | | |
| --- | --- | --- | --- | --- | --- | --- | --- | --- | --- |
|  | LVEDD (mm) | LVESD (mm) | LVEF (%) | MR grade | RAP (mmHg) | PAP (mmHg) | PCWP (mmHg) | CI (Thermo/Fick) (L/min/m2) | SvO2 (%) |
| #1 | 33 | 20 | 74 | trivial | 6 | 39/13 | 8 | 2.45/2.72 | 67.0 |
| #2 | 37 | 23 | 69 | none | 2 | 26/5 | 6 | 2.90/2.53 | 67.2 |

CI, cardiac index; LVEDD, left ventricular end-diastolic diameter; LVEF, left ventricular ejection fraction; LVESD, left ventricular end-systolic diameter; MR, mitral regurgitation; PAP, pulmonary arterial pressure; PCWP, pulmonary capillary wedge pressure; RAP, right atrial pressure; SvO2, mixed venous oxygen saturation

**Table 1** Postoperative findings of transthoracic echocardiography and right heart catheterization performed 6 months after surgery, show the favorable cardiac function of each transplanted heart.
