# Supplementary material for: Impact of bench repair for donor mitral valve before orthotopic heart transplantation: a case report
Source: Gen Thorac Cardiovasc Surg Cases. 2023 May 29;2:61. doi: 10.1186/s44215-023-00070-1 (PMC11533544; doi:10.1186/s44215-023-00070-1)
Supplement: Supplementary file 2 — Additional file 2: Supplementary Table 2. Surgical cases using donor hearts having mitral regurgitation with bench mitral valve repair before heart transplantation. [file 44215_2023_70_MOESM2_ESM.docx]

**Supplementary Table 2**. Surgical cases using donor hearts having mitral regurgitation with bench mitral valve repair before heart transplantation

| **Authors** | **Year** | **Recipient Age/Sex** | **Donor Age/Sex** | **Donor LVEF (%)** | **Surgical procedure** | **Ischemic time repair, total**  **(minutes)** | **Follow-up periods (months)** |
| --- | --- | --- | --- | --- | --- | --- | --- |
| Risher WH, et al.^2^ | 1994 | 58/M | 30/M | 50 | commissurotomy,  division of fused chordae tendineae | 10, 271 | 12 |
| Massad MG, et al.^3^ | 1996 | 57/M | 23/- | - | posterior annuloplasty | -, 219 | 18 |
| Michler RE, et al.^4^ | 2002 | 58/M | 21/M | 55 | annuloplasty | -, - | 24 |
|  |  | 54/M | 24/M | 55 | annuloplasty | -, - | 24 |
| Prieto D, et al.^6^ | 2009 | 52/M | 35/F | - | commissurotomy, posterior annuloplasty  division of fused chordae tendineae | -, 84 | 57 |
|  |  | 52/F | 17/F | - | posterior annuloplasty | -, 84 | 12 |
|  |  | 50/M | 22/F | - | artificial chorda reconstruction, partial posterior annuloplasty | -, 63 | 0.5 |
|  |  | 71/M | 47/F | - | posterior annuloplasty | -, - | 9 |
| Pawale A, et al.^7^ | 2012 | 61/M | 53/M | 55 | indentation closure, posterior annuloplasty | -, 127 | 14 |
|  |  | 58/F | 30/- | - | posterior annuloplasty | -, - | 18 |
|  |  | 58/M | 29/- | 53 | Annuloplasty | -, - | 84 |
| Okamura H, et al.^8^ | 2014 | 64/M | 43/M | - | artificial chorda reconstruction, annuloplasty | -, 303 | 3 |
| Sprengel A, et al.^9^ | 2018 | 58/M | -/- | - | annuloplasty,  plication of the posterior leaflets | 20, - | 48 |
| Fiore A, et al.^10^ | 2020 | 53/M | 47/M | 60 | annuloplasty | 22, 255 | - |
|  |  | 57/F | 64/F | 60 | artificial chorda reconstruction, annuloplasty | 25, 248 | 82 |
|  |  | 58/M | 64/M | 60 | cleft closure | 7,89 | 60 |

F, female; LVEF, left ventricular ejection fraction; M, male; MR, mitral regurgitation

**Table 2** Surgical cases of heart transplantation using donor hearts with mitral regurgitation in which bench mitral valve repair were performed prior to heart transplantation.
